# Something More about Cocaine

**Published:** 1887-05

**Authors:** 


### SELECTIONS.

### Something More About Cocaine.

Dr. Witzel is one of the most experienced dentists in Germany. That the reader may know how much credit to give to any statement of W., we will mention that he is not only an M. D., but an author, who in Germany is looked upon as the representative of scientific dentistry.

W. recommends the subgingival injection of muriate of cocaine for the painless extraction of teeth. About nine minutes after the injection into the gums of seven drops of a 20 per cent. solution—we see that the dentists in Europe are no longer satisfied with a 2 or 4 per cent. solution—an increase in the action of the heart is suddenly noticed. Then is the time to extract the tooth. The patient is said to feel the application of the forceps, and also the pulling of the tooth, but to show by no exclamation of any kind that the operation is at all painful to him.

W. made some interesting observations which fully coincided with Claude Bernard's researches. The latter, as well known, demonstrated by his experiments that cocaine in its action on the cerebral circulation was exactly the antagonist of nitrite of amyl. Under the effect of amyl, the cerebral arteries contract, anæmia of the brain develops, the arterial pressure is increased, and the face looks pale. Cocaine causes a dilatation of the vessels of the heart, hyperæmia with diminished arterial pressure sets in the encephalon, and the face appears flushed and in a general state of venous congestion.

To at once end the anæsthetic condition produced by cocaine, the inhalation of a few drops of nitrite of amyl is the best remedy. In this connection it may be well to remind dentists who may be desirous of employing subgingival injections of 20 per cent. solutions of cocaine that the remedy, at least in this strength, is not without its danger. Dr. Knabe, of Berlin, records the case of a girl, aged eleven, who, after a severe attack of scarlatina, suffered from fatty degeneration of the heart. In her case, the hypodermic injection of cocaine was recommended as a tonic to remedy the frequent fainting fits to which she was subject. Certainly the physician advising such a procedure could have had no idea of the morbid condition underlying these attacks of syncope, nor must he have possessed any knowledge of the physiological action of cocaine. Four or twelve drops—they were never able to discover the exact dose--of a four per cent. solution of cocaine were injected under the skin over the deltoid muscle of the left arm. Scarcely forty seconds later the girl took a deep breath, became deadly pale in the face, and dropped down unconscious. She never recovered consciousness, death ensuing a minute later. In this case nitrite of amyl would have been the remedy indicated.

As not all dentists are graduated physicians, they had better be careful in the administration of such strong solutions of muriate of cocaine. Nitrous oxide gas answers all their purposes, and brings less danger than any other anæsthetic.--*Editorial in Med. and Surg. Rep.*

